# Oxidative Stress and the Microbiota-Gut-Brain Axis

**DOI:** 10.1155/2018/2406594

**Published:** 2018-12-09

**Authors:** Laura Dumitrescu, Iulia Popescu-Olaru, Liviu Cozma, Delia Tulbă, Mihail Eugen Hinescu, Laura Cristina Ceafalan, Mihaela Gherghiceanu, Bogdan Ovidiu Popescu

**Affiliations:** ^1^Department of Clinical Neurosciences, Carol Davila University of Medicine and Pharmacy, 37 Dionisie Lupu Str., 020021 Bucharest, Romania; ^2^Department of Neurology, Colentina Clinical Hospital, 19-21 Stefan cel Mare Str., 020125 Bucharest, Romania; ^3^Department of Cellular and Molecular Biology and Histology, Carol Davila University of Medicine and Pharmacy, 8 Eroilor Sanitari Str., 050474 Bucharest, Romania; ^4^Laboratory of Ultrastructural Pathology, Victor Babeș National Institute of Pathology, 99-101 Splaiul Independentei Str., 050096 Bucharest, Romania

## Abstract

The gut-brain axis is increasingly recognized as an important pathway of communication and of physiological regulation, and gut microbiota seems to play a significant role in this mutual relationship. Oxidative stress is one of the most important pathogenic mechanisms for both neurodegenerative diseases, such as Alzheimer's or Parkinson's, and acute conditions, such as stroke or traumatic brain injury. A peculiar microbiota type might increase brain inflammation and reactive oxygen species levels and might favor abnormal aggregation of proteins. Reversely, brain lesions of various etiologies result in alteration of gut properties and microbiota. These recent hypotheses could open a door for new therapeutic approaches in various neurological diseases.

## 1. Introduction

The microbiota-gut-brain axis is a complex multidirectional cross-talk system between the gut microbiota, the enteric nervous system (ENS), and the brain. It acts as an adaptive interface with the environment and consists of a humoral pathway, based on the intestinal barrier, portal and systemic circulations, blood-brain barrier (BBB), and a neural pathway (via the vagus nerve) [1]. A strong interplay also exists with the neuroendocrine-immune network; therefore, the functional integrity of the axis is required for the homeostasis of several systems [1, 2].

Increasing evidence suggests that the gut microbiota is involved in several neurodegenerative disorders, such as Parkinson's disease (PD) and Alzheimer's disease (AD), as well as in acute central nervous system (CNS) injury, such as ischemic stroke [1, 2]. Interestingly, oxidative stress (OS) is also a key player in the pathogenesis of these disorders. In this review, we summarize the available data concerning potential interactions between the microbiota-gut-brain axis and CNS's oxidative stress.

## 2. The CNS Oxidative Stress: Gut Microbiota Connection—A Plausible Hypothesis

Recent experimental evidence found that, in the presence of the microbiota, the epithelial lining of the gut generates physiological levels of OS. In return, these interfere both with the composition and functionality of the microbiota (e.g., anaerobes thrive in the presence of electron acceptors) and directly with the permeability of the intestine, thus increasing the chances of xenobiotic molecules reaching the systemic circulation and the CNS [3]. The oxidative reduction potential of the gut microbiota (i.e., the tendency and capacity of the microbiota to gain electrons) influences the homeostasis of the intestinal barrier as well [3], while the brain/CNS modulates the level of OS within the intestine via the vagal cholinergic anti-inflammatory pathway [1, 4, 5]. All these may have direct or indirect (and possibly cumulating) consequences on the oxidative balance in the CNS, either by increasing the oxidant component or by interfering with the antioxidant system [2]. Therefore, one may speculate that gut dysbiosis may be both a cause and a consequence of increased levels of CNS OS [4], thus adding a new dimension to the interplay between the gut microbiota and the brain, also known as the microbiota-gut-brain axis.

## 3. Oxidative Stress and Its Role in CNS Health and Disease

### 3.1. General Considerations

OS is a type of reactive stress. As a biochemical concept, it is defined as the state of imbalance between oxidants and antioxidants, with relative excess of the former, resulting in the “disruption of redox signaling and control and/or molecular damage” [6]. Though the terminology may suggest that OS is only a disadvantageous by-product, it actually plays critical physiological roles, providing that it is maintained within a safe steady-state range (e.g., mitigating infections). At higher levels, however, OS is potentially neurotoxic, resulting in biomolecular damage, i.e., protein, lipid, and deoxyribonucleic acid (DNA) oxidation, which may result in a broad spectrum of cellular dysfunctions, culminating with cell death (Massaad and Klann, 2013, [6]). Its presence is intrinsic to the aerobic metabolism, virtually all chemical reactions involving molecular oxygen resulting in the generation of short-lived, highly unstable/reactive intermediate products, known as reactive oxygen species (ROS) [6–8].

### 3.2. Redox Reactions and the Biology of Oxidative Stress

Oxidants are chemical species able to remove and accept electrons from other atoms or electronegative atoms from other molecules [9]. Conversely, antioxidants are able to delay or prevent the effects of the oxidants, balancing the oxidative state of a system without becoming destabilized themselves [8].

The free radicals are oxidants able to remove and accept electrons from other atoms, meaning that they contain at least one unpaired electron but are stable enough to exist independently [6, 9].

The biological activity of free radicals, which includes toxic and beneficial effects, is related to their propensity for triggering reduction-oxidation or redox reactions that perpetuate in a domino-like fashion (i.e., reactions involving the transfer of an electron between two chemical species: the species gaining the electron is the “oxidant”/“oxidizing agent” which is “reduced,” and the species losing the electron is the “reductant”/“reducing agent” which is “oxidized”) [6, 8, 9].

ROS are mostly free radicals, but nonradical species are also produced. Other free radicals (either oxidant or reductant) are also generated endogenously, in physiologic conditions, as well as during interactions with exogenous factors (e.g., drugs, radiations, and xenobiotic toxins) [6, 8].

Molecular oxygen (O_2_) itself is a free radical, having two unpaired electrons which cannot be reduced simultaneously during chemical reactions, resulting in the production of ROS (e.g., superoxide anion, hydrogen peroxide, nitric oxide, perioxynitrite anion, and the hydroxyl and peroxyl radicals) [6, 8, 10].

The generation of ROS results either from physiological processes (via ROS-generating enzymes) or by interactions with potentially harmful exogenous factors [8]. Most ROS are generated as by-products of physiological processes that occur in various parts of the cell, with the main source being the mitochondria. Other sources of ROS include xanthine and flavin oxidases and cytochrome P450 [11].

The main cellular sites of ROS production are the mitochondrial complexes I (NADH-coenzyme Q oxidoreductase) and III (cytochrome c oxidoreductase), but also complex II (succinate-Q oxidoreductase) [12]. One of the major roles of the mitochondria is the production of energy in the form of adenosine triphosphate (ATP) through the process of oxidative phosphorylation (OXPHOS) [13]. This phenomenon takes place in the inner mitochondrial membrane, where four redox complexes form the electron transport chain (ETC). The production of ATP by cytochrome c oxidase (complex IV) in the final step of the ETC requires electrons that are transported from reduced nicotinamide adenine dinucleotide (NADH) and reduced flavin adenine dinucleotide (FADH_2_) with the help of complexes I and II [12]. A high quantity of O_2_ is needed in this process as it provides an efficient electron acceptor [7]. The addition of an electron to O_2_ results in the formation of superoxide (O_2_^**−**^**˙**), which is the main precursor to many ROS [14]. Under normal conditions, the levels of superoxide resulting from the ETC are decreased by antioxidant enzymes such as manganese superoxide dismutase (SOD) and copper-zinc SOD that convert superoxide to hydrogen peroxide (H_2_O_2_) and O_2_, the former being afterwards transformed to water through the action of catalase and glutathione peroxidase [15, 16]. However, this process is not perfect and the passage of electrons through the ETC also results in electron leakage and the subsequent formation of low levels of superoxide [17]. The addition of an electron to superoxide leads to the generation of ROS such as H_2_O_2_ and hydroxyl anion (^**−**^OH)—the higher the amount of oxygen is, the higher is that of superoxide resulting in greater ROS production [7]. Hydroxyl radical (HO**˙**) is very reactive, and its formation depends not only on the amount of oxygen but also on that of iron or copper ions that serve as catalytic factors in what is known as the Fenton reaction (O_2_^**−**^**˙** + H_2_O_2_ → HO**˙** + OH^**−**^ + O_2_) [12].

Though the ETC is the major source of ROS production in the cell, it is not the only one. The action of monoamine oxidases inside the outer mitochondrial membrane leads to H_2_O_2_ formation, while the transfer of electrons from NADPH in the endoplasmic reticulum results in more ROS production [12]. Lipoxygenases, a class of enzymes that oxidize esterified and free polyunsaturated fatty acids (PUFA), also catalyze the transformation of arachidonic acid leading to hydroxyl radical and superoxide formation [18].

Peroxisomes contain various enzymes involved in the metabolism of lipids that generate ROS as part of their normal catalytic cycle. Beta-oxidation of fatty acids and the actions of glycolate oxidase and xanthine oxidase produce superoxide and H_2_O_2_ [12]. Xanthine oxidases are also located in the cytoplasm, where they are involved in the metabolism of purines, a process that leads to superoxide formation [19].

ROS are also generated by leucocytes and microglia in the brain. When these cells are activated, they increase oxygen consumption in the process known as “respiratory burst” and use various enzymes in reactions that result in the production of ROS such as H_2_O_2_ or hypochlorous acid (HOCl) [12, 20].

### 3.3. The Physiological Roles of Oxidative Stress

The free radicals generated by the mitochondrial ECT are efficiently used by the innate immunity for mitigating infections [9]. OS also has beneficial/homeostatic roles in the CNS, with ROS/RNS playing important roles in several processes such as the growth of hippocampal progenitor cells, synaptic plasticity, and axonal path finding [9, 21–23]. Moreover, free radicals are also involved in cellular redox signaling and other prosurvival pathways, mediated by “redox sensors” that modulate the expression of certain enzymes, and are kept under control by soluble and insoluble “redox sinks” (e.g., glutathione and thioredoxin—see below) [10]. These redox-active proteins (sensors and sinks) may undergo rapid, reversible, and gradual oxidation of their numerous cysteine residues, buffering the free radicals and concomitantly allowing for an accurate perception of the intracellular levels of ROS (and thus for the fine tuning of the responses) [4, 8].

Thus, in physiological conditions, ROS are involved in interconnected processes such as inflammation, signaling transduction pathways, the immune response, and apoptosis. There is a dual relationship between OS and inflammation: OS can be induced by inflammatory responses, and inflammation can be triggered or enhanced by ROS through activation of nuclear factor-kappa B (NF-*κ*B), which controls the expression of many genes, including some involved in inflammatory responses leading to the production of various cytokines [24]. Leucocytes induce OS in the process of phagocytosis by generating ROS in reactions catalyzed by NADPH oxidase, superoxide dismutase, and myeloperoxidase (MPO) [12]. MPO is a lysosomal enzyme that generates HOCl used as a powerful oxidative agent against pathogens [20]. ROS also seem to be involved in various signal transduction pathways playing a role in intracellular signaling and regulation in regard to cytokine and growth factor signaling, nonreceptor tyrosine kinases, protein tyrosine phosphatases, serine/threonine kinases, and nuclear transcription factors [14].

### 3.4. The Roles of Oxidative Stress in CNS Disorders

OS exerts most of its deleterious effects by inducing lipid peroxidation and by damaging nucleic acids and proteins. The main targets of lipid peroxidation are PUFA, such as arachidonic acid and linoleic acid, lipids that are found in abundance in the cell membrane [25]. The addition of hydrogen from ROS to PUFA leads to the formation of lipid peroxyl radical which interacts with another PUFA that then reacts with oxygen and forms another lipid peroxyl radical, generating a chain reaction [25]. Since the hydroxyl radical has a very high chemical reactivity, it is the most effective ROS in inducing lipid peroxidation thus producing significant damage to the neuronal membrane. The cellular levels of hydroxyl radical depend on the amount of available oxygen, but also on those of iron and copper that catalyze the previously described Fenton reaction in which hydroxyl radical results from superoxide and H_2_O_2_ [12].

Nucleic acids like ribonucleic acid (RNA), nuclear DNA, or mitochondrial DNA (mtDNA) are targets of OS. The hydroxyl radical can permanently damage the DNA by inflicting injuries to purines, pyrimidines, and deoxyribose, but most notably, it is mtDNA that is prone to oxidative damage since mitochondria are the main site of ROS production and mtDNA is in direct contact with ROS [13, 26].

Protein oxidation may lead to changes in their function such as activation, inactivation, or gain of a new function, depending on the specific oxidative modification taking place with consequences on various signal transduction pathways [26].

There are multiple factors that make the brain particularly susceptible to OS. Most notably, the brain has a high rate of oxygen use, amounting to about 20% of total oxygen consumption, even though it represents only 2% of the body's total weight [7]. As we have previously discussed, the generation of ATP through the ETC leads to electron leakage and superoxide formation with subsequent ROS production. Since the brain utilizes large quantities of oxygen, it also generates a significant amount of ROS. Moreover, the brain has regions with high levels of iron which is used to generate even more hydroxyl radical. This increase in ROS production is met by a greater concentration of PUFA making the brain more susceptible to OS [27].

Besides OS occurring as a part of physiological processes that may be more or less related to normal aging, it is important to note that disease states lead to greater production of ROS and subsequent oxidative damage. As such, OS has been involved in the pathology of various chronic disorders of the brain such as AD [28, 29], PD [30], Huntington's disease [25], amyotrophic lateral sclerosis [31], multiple sclerosis [25], and depression [32], as well as in acute damage that occurs in stroke [33] or traumatic brain injury [34].

### 3.5. The CNS Antioxidant Metabolism and Gut Microbiota Interference

The CNS is highly susceptible to OS, and chronic OS is a putative mechanism in many of its diseases. This is explained by its narrow redox homeostatic window, the proper functioning of the CNS generating and requiring high levels of ROS (i.e., strong oxygen demand, with high oxidative metabolism and extensive use of ROS and other reactive species for intra- and intercellular signaling) [9, 11]. Other particularities contributing to the high susceptibility of the CNS to OS include its high content of redox-active transition metals (e.g., iron and copper) and PUFA (which are prone to peroxidation) and the presence of autooxidating neurotransmitters [9, 11, 35].

Complex gut microbiota microbe-microbe and microbiota-host interactions may also influence the oxidative state of the CNS, directly and indirectly, by interfering both with the level of ROS (endogenous and exogenous) and with the antioxidant system [1, 2, 4]. These mechanisms are mostly speculative but are pertinent to the hypothesize that the oxidative state of the CNS could be regulated by the microbiota via the production of various metabolites (i.e., absorbable vitamins, short-chain fatty acids (SCFA), polyphenols, and highly diffusible antioxidant and oxidant gases), optimization of dietary energy harvest, regulation of the permeability of the intestinal barrier and BBB, immune system modulation, and prevention of extensive colonization by pathogenic microbes ([1, 2], Ravcheev and Thiele, 2012, [4, 36, 37]). The microbiota also produces considerable amounts of CNS neurotransmitters (e.g., dopamine, serotonin, and gamma-amino butyric acid) which modulate the local activity of the ENS and may correlate with their respective levels within the CNS, depending on the intestinal and BBB permeability [9]. Moreover, the microbiota may also produce neurotoxic and potentially neurotoxic substances (such as lipopolysaccharides and amyloid proteins) which may reach the CNS via the systemic circulation or the vagus nerve, promoting microglia activation and neuroinflammation, increasing the CNS production of ROS and/or making neurons more susceptible to OS [9].

The antioxidant metabolism of the CNS is relatively modest, but tightly regulated [8, 9]. The enzymatic antioxidants include SOD, which reduces the superoxide anion to O_2_ and H_2_O_2_ and is essential for cell survival; glutathione and glutathione peroxidases, which are selenium-dependent and selenium-independent isoenzymes that use glutathione to catalyze the reduction of H_2_O_2_ and lipid peroxides; peroxiredoxins which are thiol-specific peroxidases found in the cytoplasm, nuclei, mitochondria, peroxisomes, and lysosomes that catalyze the reduction of hydroxyperoxides (including H_2_O_2_ and peroxynitrite); and catalase which converts H_2_O_2_ to water and oxygen, using iron or manganese as a cofactor, but has low CNS expression (i.e., 50 times lower than in hepatocytes) and minor roles at steady-state levels [2, 9, 11].

The presence of moderate levels of ROS activates transcription factors that increase the antioxidant potential (/defense), thus priming the CNS for exogenous OS and increasing the chances of cell survival [9]. Glutathione has a low expression in the CNS (about half of that found in other tissues) [11]. In its reduced form, it reacts nonenzymatically with free radicals and functions as the electron donor for the reduction of peroxide by glutathione peroxidases, resulting in glutathione disulfide. The latter can be regenerated to glutathione by a reductase which transfers electrons from NADPH (Tse, 2015, [11]). The low CNS levels of glutathione may limit glutathione peroxidase 4 activity, thus possibly explaining the high neuronal susceptibility to iron-related programmed cell death (i.e., ferroptosis) [9, 11]. However, glutathione peroxidase 1 is one of the most important antioxidant systems in the CNS. It is expressed in the microglia (but not in neurons) and is upregulated in response to injury, having a cytoprotective effect. Peroxiredoxin-thioredoxins are a NADPH-dependent enzymatic system which is highly expressed in neurons. It is involved in redox-transducing signaling and may be required for the efficient metabolism of H_2_O_2_ [9]. Another antioxidant system expressed by CNS cells consists of peroxiredoxins, which are responsible for the reduction of up to 90% of mitochondrial H_2_O_2_ and almost all cytoplasmic H_2_O_2_ (Tse, 2015, [11]). Also, an important cytoprotective pathway (arguably the most important) is the Kelch-like ECH-associating protein 1-nuclear factor erythroid 2-related factor 2-antioxidant response element (Keap1-Nrf2-ARE), which is highly expressed in neurons. It responds to both physiological and pathological/xenobiotic OS by modulating the expression of SOD, thioredoxin, peroxiredoxins, and glutathione peroxidases [4, 9]. The “redox sensor” protein NF-*κ*B is also expressed in the CNS, activating the transcription of antiapoptotic proteins and inhibiting caspase-dependent cell death [4, 9, 11]. Higher levels of ROS, however, are proapoptotic, inhibiting the binding of NF-κB to the DNA [8, 11].

#### 3.5.1. Nitrosative Stress and Antinitrosative “Defense”

The reactive chemical species generated by the activity of NADPH oxidase (Nox) are categorized as reactive nitrogen species (RNS) and may result in the so-called nitrosative stress (NS). NS typically accompanies OS [2, 6, 8].

Nitric oxide (NO) (i.e., the endothelium-derived relaxing factor) is another free radical and also the main neurotransmitter of the nonadrenergic noncholinergic ENS. It is a highly diffusible short-lived gas, synthesized endogenously from L-arginine and oxygen by various nitric oxide synthases (NOS), using nicotinamide adenine dinucleotide phosphate (NADPH) as a cofactor [2]. It functions as a signaling molecule and neuroprotector at low levels, resulting in neurotoxic RNS and OS/NS with harmful neuroinflammatory repercussions at higher levels [2]. Its neuroprotective effects are mediated by the nitrosylation of the N-methyl D-aspartate (NMDA) receptors and caspases, limiting excitotoxicity, and apoptosis [2, 6]. The mechanism of NO toxicity is related to its interaction with other ROS resulting in the generation of highly reactive peroxynitrite, hydrogen peroxide, hypochlorite ions, and hydroxyl radical. Elevated NO levels also downregulate the secretion of brain-derived neurotrophic factor, reducing the neuronal survival and synaptogenetic processes [2, 6, 11]. At gastrointestinal levels, NO is released by ENS inhibitory motor neurons via the activation of NMDA receptors, as well as by infiltrating neutrophils and monocytes. In the CNS, it is generated in nanomolar amounts from L-arginine by the endothelial and neuronal NOS (eNOS and nNOS, respectively), while inducible NOS (iNOS) secretes higher, neurotoxic levels, in response to proinflammatory stimuli [2, 6]. The main producer of NO is the gut microbiota via the reduction of gastric nitrate and nitrite and denitrification. A higher nitrate intake may increase the production of nitrite, NO, and ammonium (NH_3_) by certain salivary and also intestinal bacteria using L-arginine-dependent and L-arginine-independent pathways. This results in higher levels of NO within the intestinal tract and the CNS (e.g., NO is absorbed from the intestinal tract and may be scavenged by erythrocyte hemoglobin, reaching the CNS via the systemic circulation), with potentially deleterious consequences [2].

#### 3.5.2. Other CNS Antioxidants

Molecular hydrogen (H_2_, dihydrogen) is another highly diffusible bioactive gas. It has antioxidant properties, reducing hydroxyl radicals and possibly peroxynitrite (ONOO^−^), but not other reactive ROS/RNS, and raised interest over the past years due to its efficiency in ameliorating several disease processes associated to OS [36, 37]. Humans do not directly produce it, but the average microbiota generates about 1 liter per day during the process of fermentation [36–38]. The hydrogen-producing bacteria include anaerobic cocci, members of the Enterobacteriaceae family and certain strains of the Clostridium genus. These are usually accompanied by symbiotic counterparts that consume H_2_ (e.g., methanogens, sulfate-reducing bacteria, and acetogens bacteria); thus, the production of H_2_ varies between individuals and within the same individual, in relation to the composition of the microbiota and the diet. Since H_2_ is produced by gut bacteria, but not humans, it is plausible to consider that gut dysbiosis may result in low H_2_ production, limiting the availability of the gas to the CNS neurons and increasing their susceptibility to OS-related disorders [38].

## 4. The Gut-Brain Axis in Neurodegeneration

There is a two-way connection between our gut and our brain, which is important not only for the physiology of the digestive system but for good brain health as well. The ENS is the largest component of the autonomous nervous system, with a number of nervous cells similar to the spinal cord, and has integrative activity [39]. The vast majority (90%) of vagus nerve fibers are afferent, making the gut a possible large access door to brainstem and CNS [40].

**Figure 1:**
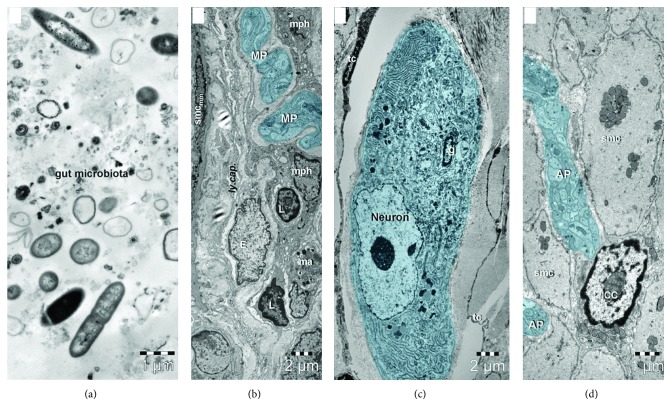
Transmission electron microscopy images of the (a) gut microbiota, (b, c) submucosal Meissner plexus, and (d) myenteric Auerbach plexus from the small intestine of 6-month-old mouse. (a) Various types of bacteria, nanoparticles, and vesicles with diverse dimensions from intestinal lumen. (b) Nerve endings of the submucosal plexus (MP—Meissner plexus) run along a lymphatic capillary (ly.cap.) between macrophages (mph), mast cells (ma), lymphocytes (L), and smooth muscle cells of muscularis mucosae (smc_mm_). (c) A neuron and an enteric glial cell (g) from the submucosal Meissner plexus are surrounded by telocytes (tc). (d) Nerve fibers from the myenteric Auerbach plexus (AP) in contact with an interstitial Cajal cell (ICC) between smooth muscle cells (smc) of the muscularis.

Most neurodegenerative disorders are proteinopathies, meaning that they are associated with intraneuronal protein misfolding and aggregation. OS is another shared pathogenetic factor; however, the etiopathogeny of these diseases is incompletely understood and disease-modifying treatments are not available. Heiko Braak has made an important contribution when he unravelled the presence of aggregated *α*-synuclein in submucosal Meissner's and myenteric Auerbach's plexuses in the stomach and gut in PD patients, hypothesizing that the misfolded protein pathology might start in the intestine and ascend transynaptically to CNS neuron populations, resulting in neurodegeneration [Braak et al., 2006]. Considering the proximity of ENS neurons to the intestinal lumen, the gut microbes were considered as plausible triggering factors—for the morphologic relationship of nerve endings with other cells and structural elements (lymphatics, smooth muscle cells, and immune-competent cells) and gut microbiota (please see Figure 1). Later on, accumulating data suggested that gut microbiota might influence the aggregation and propagation of *α*-synuclein and Friedland and Chapman proposed the term *mapranosis* (i.e., microbiota-associated proteopathy and neuroinflammation) in order to describe the influence of the microbiota on the brain [41].

## 5. Oxidative Stress and the Microbiota-Gut-Brain Axis in Parkinson's Disease

Sporadic PD is the second most common neurodegenerative disorder, after AD. It is a multifactorial disease, involving selective loss of central and peripheral aminergic neurons associated with intracytoplasmic aggregation of misfolded *α*-synuclein, i.e., the so-called Lewy bodies and Lewy neurites [42, 43]. Accumulating evidence supports the initial hypothesis of Braak, suggesting that the pathogenic process begins in the gut, progressing towards the CNS via the vagus nerve. Thus, the gut microbiota and gut dysbiosis are highly plausible contributing environmental factors to the development and progression of PD, as suggested by recent animal findings and indirect human data [11, 42–44].

The motor symptoms, which are still considered the hallmark of PD, become prominent in the later stages of evolution and are related to the selective loss of dopaminergic neurons in the substantia nigra pars compacta (SNpc) [11, 42]. The presence of OS is supported by the finding of lower SNpc glutathione levels and higher iron, H_2_O_2_, and lipid peroxidation, which may result in increased production of the highly toxic hydroxyl radicals and subsequent neuronal death [11, 45]. The particular susceptibility of SNpc neurons may also be related to the metabolism of dopamine itself. This involves monoamine oxidase enzymes which catalyze a deamination reaction generating hydrogen peroxide and ammonia. Neuronal activity (i.e., oxygen saturation) induces OS and dopamine oxidation results in several H_2_O_2_ molecules and electrophilic aldehyde metabolites. Dopamine reacts with molecular oxygen, forming dopamine semiquinone radical, which reacts with another dioxygen molecule to form dopamine quinine. Redox transition metals increase this reaction, and dopamine quinones may interact to form semiquinones. The oxidation products of dopamine metabolism may also enter redox cycling, forming superoxide anion and hydroxide peroxide, which contribute to the pathogenesis of PD [9] The mechanism of neuronal death in PD is incompletely understood, but the available evidence suggests that mitochondrial dysfunction and OS are key pathogenic pathways [11, 46]. Mitochondrial respiratory chain dysfunction (especially complex I deficiency) is present in PD and results in the production of excessive ROS, leading to apoptosis [11, 44, 46]. This is also supported by the cytotoxic effect induced on dopamine neurons by complex I inhibitors, such as 1-methyl-4-phenyl-1,2,3,4-tetrahydropyridine (MPTP) [11, 42, 43, 46]. The monogenic PD cases related to *α*-synuclein, parkin, phosphatase, and tensin homolog-induced putative kinase (PINK) also show mitochondrial dysfunction and high OS levels, supporting these as plausible mechanisms [11, 46]. The role of *α*-synuclein is still being debated, but a recent study found a conformationally distinct *α*-synuclein aggregate that induces mitochondrial damage and mitophagy [44]. Moreover, increased levels of OS and decreased free radical scavenger capacity exacerbate *α*-synuclein aggregation in animal models [47]. Mitochondria are bacterial endosymbionts which maintain some pathogen-associated molecular patterns and release damage-associated molecular patterns, triggering innate immunity responses, which may result in even higher levels of OS [44, 46]. Considering the above, it has been hypothesized that some members of the gut microbiota may produce toxins (/antibiotics) targeting the mitochondria of the ENS and CNS that could result in subsequent neurodegeneration [46].

As already discussed, decreased production of H_2_ by the microbiota has been proposed as an environmental factor which may interfere with the development and subsequent evolution of several diseases, including sporadic PD [38]. In a rat model of PD, 50%-saturated H_2_ drinking water was successful in preventing nigrostriatal degeneration [36, 37]. H_2_-water also prevented neuronal loss and reduced OS markers in the substantia nigra of a MPTP mouse model [37]. In humans, a pilot placebo-controlled, double blind, randomized trial found H_2_-water to be beneficial, improving the motor ratings of PD patients [36, 37]. Though it is tempting to speculate that gut dysbiosis may result in low H_2_ production, with a negative impact on PD evolution, further investigation is needed [38].

## 6. Oxidative Stress and the Microbiota-Gut-Brain Axis in Alzheimer's Disease

AD is the most common neurodegenerative disorder [48]. It is characterized by a progressive impairment in episodic memory and other cognitive domains, progressing towards dementia [49]. The neuropathology hallmarks of AD are cerebral extracellular amyloid plaques embodying amyloid-*β* (A*β*) that aggregates and adopts a *β*-sheet structure and intracellular neurofibrillary tangles comprising hyperphosphorylated tau protein [48, 50]. Brain atrophy emerges as a consequence of synaptic degeneration and neuronal death, notably involving the hippocampus [48]. Decades of research failed to fully elucidate its etiopathogenesis, whereas preventive or disease-modifying therapies are still missing. New insights into the mechanisms of AD are required in order to conceive effective treatments.

OS is currently regarded as a key process in the pathogenesis of AD. The high energetic demands of the nervous system enhance exergonic oxidative processes, subsequently exposing the neurons to ROS [49, 51]. Considering the substantial PUFA able to interact with ROS and low levels of glutathione responsible for impaired clearance of free radicals, neurons are particularly vulnerable to OS [11, 49, 52].

The role of oxidative damage in the pathogenesis of AD is reflected by altered activity of antioxidant enzymes (SOD, catalase) and increased levels of OS biomarkers (malondialdehyde, 4-hydroxynonenal, and F2-isoprostane protein are markers of lipid oxidative damage; protein carbonyls and 3-nitrotyrosine are products of protein oxidation, whereas 8-hydroxydeoxyguanosine reflects nucleic acid oxidation) in the blood and cerebrospinal fluid of patients with AD [11, 52, 53]. Moreover, the amount of oxidative markers is directly proportional to the degree of cognitive impairment [54] and brain weight [52]. It is noteworthy that 8-hydroxydeoxyguanosine excess in the parietal cortex precedes the pathognomonic histopathological abnormalities of AD by decades, whereas patients with mild cognitive impairment have elevated levels of malondialdehyde, 4-hydroxynonenal, F2-isoprostane protein, and protein nitration products in the brain [52]. These findings suggest that OS is not merely a collateral event in the pathogenesis of AD, but rather an early prominent process. Inflammation, which is one of the key elements of AD pathogenesis [55], is an important OS trigger, and there is still no intervention to alleviate its effects, since it activates both favorable and unfavorable signaling pathways [56].

There is a mutual relationship between OS and A*β* production and aggregation in AD—OS enhances A*β* deposition, whereas A*β* triggers oxidative reactions [11]. Interestingly, A*β* aggregates facilitating OS are confined not only to extracellular regions but also to cellular organelles such as Golgi apparatus, endoplasmic reticulum, and mitochondria with emergent mitochondrial dysfunction [11]. Tau pathology is also linked to OS. Cells with overexpressed tau protein exhibit decreased NADH-ubiquinone oxidoreductase activity and mitochondrial dysfunction that generate ROS [11].

Multiple mechanisms underlie OS, particularly mitochondrial dysfunction, metal accumulation, hyperphosphorylated tau protein, and inflammation [52]. Mitochondrial dysfunction is promoted by defective ETC enzymes (i.e., cytochrome oxidase), mitochondrial DNA mutations, and inactivation of antioxidant mitochondrial enzymes (i.e., SOD) and leads to significant ROS production and scarce energy stores in hippocampal neurons of AD patients [11, 52]. Metal accumulation has been found in the hippocampus and amygdala of AD patients; copper and iron generate ROS by binding to A*β*, whereas zinc is a component of amyloid plaques [52]. Hyperphosphorylated tau protein burden is proportional to ROS levels [52]. Since neurofibrillary tangles exhibit decreased levels of 8-hydroxydeoxyguanosine despite marked OS, tau phosphorylation is supposedly involved in cytoprotection against oxidative damage [52]. Inflammation also triggers oxidative reactions [52]. A*β* activates microglia and astrocytes that release cytokines, chemokines, and ROS [52]. Provided that OS contributes to A*β* and tau pathology in AD, antioxidants are potential effective treatments by lowering ROS and protecting nervous cells from oxidative insult [52].

The intestinal microbiome seems to play a significant role in AD pathogenesis, as suggested by gut microbiota shifts towards proinflammatory bacteria in transgenic APP/PS1 mice [57] and declining amyloid plaque deposition and neuroinflammation (as indicated by reduced plaque-localized gliosis and modified microglial phenotype) in the same murine model treated with long-term broad spectrum combinatorial antibiotics [58].

Altered gut microbiota enhances the cerebral aggregation and deposition of A*β* by immune, metabolite-mediated, endocrine, and neural pathways [50]. Amyloid proteins produced by bacterial populations (i.e., microbial amyloid) activate the innate immune system, subsequently inducing a response that entails TLRs and CD14 and elicits underrecognition of misfolded A*β* with impaired A*β* clearance [41]. A concomitant humoral reaction involves proinflammatory cytokine activation with ensuing disruption of intestinal and blood-brain barriers [50]. Microbial metabolites such as hydrogen sulfide, trimethylamine, and SCFA are likely to be involved in AD pathogenesis [41]. Reduced plasmatic levels of enteric hormones with impaired signaling pathways have been reported in AD [59]. Ghrelin prevents synaptic degeneration and memory loss, leptin acts as a neuroprotective factor against A*β* toxicity by directly regulating *γ*-secretase-mediated amyloidogenic pathway, and glucagon-like peptide 1 diminishes A*β* load, whereas glucose-dependent insulinotropic polypeptide exerts neuroprotective effects [59]. Microbiota modulation by either probiotic mixtures (lactic acid bacteria and bifidobacteria) or anti-inflammatory bacterial metabolites such as SCFA increases the synthesis of enteric hormones and counteracts the progression of AD in animal models [59].

Regulatory interventions on microbiota also improve proteolytic pathways usually impaired in AD [59]. Declining hippocampal apoptosis and ubiquitin conjugate levels such as p27 and p53 in AD mice following administration of probiotics indicate enhanced proteasome functionality [59]. Facilitation of autophagy as proved by diminished cathepsin B activity (lysosomal enzyme associated with amyloid plaques in AD) as opposed to cathepsin L activity (lysosomal enzyme that amplifies *α*-secretase-mediated nonamyloidogenic pathway) also occurs [59].

Another suggested mechanism underlying microbiota-mediated cerebral amyloid accumulation is *cross-seeding of microbial amyloid* (i.e., promotion of amyloid-misfolded aggregates from one protein to a different one) via the autonomic nervous system in a manner similar to prion propagation [41, 60]. Additionally, distinct amyloid conformers interacting with cellular targets could induce different toxicities that might explain the existence of various AD phenotypes [61].

Provided that gut microbiota dysbiosis interferes with the bidirectional signaling of the gut-brain axis, modulating it through dietary or microbiotic interventions is presumably a potential therapeutic strategy [50].

Intestinal microbiota regulates several homeostatic functions. Recent data suggest that certain bacterial strains such as Lactobacilli are able to promote generation of physiological levels of ROS within epithelial cells [4]. Apart from their antimicrobial role, some ROS species such as H_2_O_2_ produced in this manner are involved in critical signaling pathways [4]. The Keap1/Nrf2/ARE pathway deals with OS by expressing genes that encode antioxidant and detoxification enzymes, hence assuring the intracellular redox hemostasis and cytoprotection [4]. Keap1/Nrf2/ARE also enhances antioxidant reactions and cellular repair mechanisms in inflammatory states [4]. NF-*κ*B is another signaling pathway that generates proinflammatory cytokines and antibacterial factors [62]. Nevertheless, it is the concentration of ROS in the cell that provides the shifts towards beneficial or unfavorable processes (i.e., cell proliferation differentiation, cytokine release, cell death by apoptosis, or activation of the innate immune system) [62]. Both under- and overproduction of ROS elicit damaging cellular reactions, so maintaining a redox balance is essential [62]. Since altered microbial population dysregulates ROS generation causing alteration of the resident microbiota profile, providing an anti-inflammatory milieu through probiotics is seemingly helpful [62].

## 7. Oxidative Stress and the Microbiota-Gut-Brain Axis in Ischemic Stroke

Ischemic stroke is an acute life-threatening condition and a leading cause of death and long-term neurological disability worldwide, with up to 40% of stroke cases not expected to recover autonomy. It results as a consequence of the interruption or severe reduction of the blood flow in the cerebral arteries, leading to oxygen and glucose deprivation, and the accumulation of waste metabolites in the affected area, with harmful effects on energy-dependent neuronal processes. The affected region of the brain is defined by two major areas of injury: the core and the surrounding region, known as the penumbra. Within the core, the brain tissue undergoes irreversible cellular damage resulting in almost instant neuronal death; the penumbra is a dysfunctional but still viable cerebral tissue characterized by moderate hypoperfusion and preserved structural integrity; it may evolve either towards necrosis or towards recovery [33].

In the setting of an acute stroke, the ensuing cerebral ischemia leads to anaerobic glycolysis and lactic acidosis subsequently promoting a prooxidant effect by increasing H^+^ concentrations and excessive production of ROS. This is a common consequence of several types of brain insults, representing a fundamental mechanism of cerebral damage in stroke [63]. Along with the accumulation of excessive levels of ROS, the ROS scavenging capacity is decreased, presumably due to an impairment of the antioxidant defense systems. Experimental studies have shown that the expression of SODs, CATs, GPx, and glutathione is significantly reduced in animal models of stroke [64]. Furthermore, although rapid restoration of blood flow in the ischemic brain is essential to prevent neuronal death in the hypoperfused area surrounding the ischemic core, the required recanalization interventions may also result in tissue damage known as “reperfusion injury.” During ischemia and reperfusion conditions, the accelerated ROS generation exceeding the endogenous antioxidant capacity is one of the main hallmarks in the pathogenesis of brain tissue destruction [65].

The ischemic cascade begins with depletion of cellular energy by failure of ATP synthesis. This adversely affects Na^+^/K^+^-ATP-ase pump and Ca^2+^ pump, resulting in plasma membrane depolarization and critical rise in intracellular calcium concentration, respectively. Furthermore, the initial calcium influx triggers a secondary intracellular toxic calcium overload. An important role in ischemic injury is also played by glutamate which is crucial for neuron degeneration when it acts as a toxic excitatory neurotransmitter. Moreover, activation of glutamate receptors (mostly NMDA) facilitates influx of calcium into neurons leading to excitotoxicity. During excitotoxicity, increasing the mitochondrial calcium concentration leads to the excessive production of ROS [63].

ROS have significant vascular effects ultimately influencing cerebral blood flow. Considering particular concentrations along with direct or indirect pathogenic pathways, the same free radical exerts divergent reactions. For instance, the superoxide is known for its biphasic induced effect on vascular tone which is remarkably complex, causing arterial relaxation if superoxide is produced from xanthine while excessive levels of superoxide in the presence of NADPH or high NADH concentrations cause cerebral arterial contraction. The effect of H_2_O_2_ on vascular tone has been largely investigated. In mice, in vivo application of H_2_O_2_ induces basilar artery dilation [33]. Nonetheless, high levels of H_2_O_2_ can produce vasoconstriction followed by vasodilatation. Low concentration of ONOO^−^ induces vasodilatation of cerebral arterioles in vivo via activation of potassium channels. An animal model study reported that low concentrations of ONOO^−^ produced contraction of the posterior cerebral artery following middle cerebral artery occlusion but higher concentrations induced vasodilatation and loss of myogenic activity [66].

ROS indirectly influence the platelet activity by reducing the antiplatelet properties of the endothelium as well as scavenging NO. Moreover, O_2_^−^ in particular may induce spontaneous aggregation presumably as a consequence of reduced bioavailability of NO as a potent inhibitor of platelet activation. Experimental data have shown that platelets themselves generate ROS via enzymatic systems (NADPH, NOS, XO, and phospholipase A2) [67].

ROS have substantial cellular effects in stroke resulting in neural tissue demise and neuronal death. Two of the major consequences of ROS-induced brain injury are lipid peroxidation and protein denaturation. ROS also promote DNA modification derived by two separate mechanisms including oxidative alterations and endonuclease-mediated DNA fragmentation. Furthermore, following ROS-induced release from the mitochondria, cytochrome c forms the apoptosome and activates caspases that can cleave nuclear DNA repair enzymes increasing the oxidative DNA lesions. More than that, ROS can be involved in apoptotic pathways also by activating caspase-activated DNase which can cleave DNA resulting in apoptosis [68].

In addition to the aforementioned mechanisms, ROS mediate BBB dysfunction directly by oxidative damage, tight-junction alteration, cytoskeletal reorganization, and matrix metalloproteinases activation. It is well known that BBB is composed of the endothelial cells of the capillary wall, tight junctions among endothelia, basal membrane, pericytes, and astrocyte endfeet encircling the capillary. *In vitro* exposure of human umbilical vein endothelial cells to H_2_O_2_ induces redistribution of occludin and dissociation from zonula occludens-1 (ZO-1) [69]. Moreover, exposure of bovine pulmonary artery endothelial cells to H_2_O_2_ activates focal adhesion kinase leading to actin cytoskeleton reorganization and subsequently to increased permeability [70].

Over the last few years, considerable progress has been made regarding the role of the gut microbiota in stroke. However, plenty of concerns have remained unanswered. Following cerebrovascular events (either ischemic or hemorrhagic stroke), up to 50% of patients develop gastrointestinal complications consisting of dysphagia, gastrointestinal haemorrhage, constipation, or bowel incontinence. These result in poor patient outcomes, including delayed patient rehabilitation, increased mortality rates, and degrading neurologic function [71].

A recent experimental study using a middle cerebral artery occlusion mouse model analyzed both the intestinal bacterial biomass and composition demonstrating that stroke promotes intestinal barrier breakdown and substantial microbiota alteration. The results also strongly indicated systemic dissemination of gut bacteria [72].

It was recently reported that the brain infarct volume after transient middle cerebral artery occlusion was decreased by 60% when intestinal microbial diversity was reduced in amoxicillin/clavulanate-treated mice [73]. This study also emphasized the trafficking of intestinal T cells to the meninges. The remodeling of intestinal microbiota after stroke defines gut dysbiosis and influences immunological changes [73]. As we mentioned before, neuroinflammation and OS are closely involved in cerebral ischemia-reperfusion injury. OS induces inflammation, while inflammation causes damage through OS. Previous researching evidence established that inflammation is a decisive step in the pathophysiology of ischemic stroke. Moreover, numerous studies have indicated that neuroinflammation ensuing stroke is a determinant factor of acute outcome and long-term prognosis for ischemic stroke [74]. Therefore, various experimental approaches have explored the therapeutic potential of immunomodulation. Commensal intestinal bacteria influence the host immune system and subsequently the disease mechanisms in several organs, including the brain. Intestinal commensal microbes appear to be a potent regulator of lymphocyte populations, including regulatory T (Treg) and *γδ* T cells, both of which are involved in cerebral ischemic injury. *γδ* T cells represent a considerable lymphocyte population with innate immune properties which can exacerbate ischemic brain injury by secreting IL-17 and generating chemotactic signals for neutrophils and monocytes. Proinflammatory cytokines inhibit brain repair due to an increased production of ROS-generating OS. On the other hand, Treg cells contribute to neuroprotection by secreting the anti-inflammatory cytokine IL-10 and downregulating postischemic inflammation [75].

Summarizing, extensive stroke lesions lead to gut dysbiosis which consecutively affects stroke outcome via changes in T cell homeostasis, inducing a proinflammatory response and OS. Therapeutic transplantation of fecal microbiota in models normalizes brain lesion-induced dysbiosis and improves stroke outcome. Therefore, a target of stroke-induced systemic alterations is the gut microbiome, which is an important determinant with substantial impact on stroke outcome [63].

## 8. Conclusions

The CNS is highly susceptible to OS and chronic OS is involved in many CNS diseases. This may be explained by certain particularities: the CNS has a strong oxygen demand with a high oxidative metabolism but a modest endogenous antioxidant defense, it extensively uses ROS/RNS and other reactive species for intra- and intercellular signaling, it uses interneuronal signaling pathways that generate ROS (e.g., glutamate and calcium transients), it is abundant in redox-active transition metals (e.g., iron and copper) and PUFA (which are prone to peroxidation), it has a high glucose metabolism and high mitochondria activity, and it has autooxidating neurotransmitters.

The complex microbiota-host cross-talk occurring via the microbiota-gut-brain axis may influence the OS of the CNS, directly and indirectly, by interfering both with the local level of ROS/RNS and with the CNS antioxidant system. Among these, the production of potentially neurotoxic molecules, such as lipopolysaccharides, amyloid proteins, or antibiotics, which may reach the CNS via the systemic circulation or the vagus nerve, promoting microglia activation and the production of ROS and OS, should be further explored. Identification of microbiota biomarkers related to deleterious CNS OS also deserves further attention. The microbiota-gut-brain axis opens a gate for new therapeutic approaches of various neurological conditions.
